# Merkel cell polyomavirus in lung carcinogenesis: evidence and controversy

**DOI:** 10.3389/fonc.2025.1612605

**Published:** 2025-09-24

**Authors:** Changlong Zhou, Liang Lu, Maohong Hu, Tetsuro Suzuki, Xianfeng Zhou

**Affiliations:** ^1^ Department of Public Health, Jiangxi Province Hospital of Integrated Chinese and Western Medicine, Nanchang, China; ^2^ Jiangxi Provincial Health Commission Key Laboratory of Pathogenic Diagnosis and Genomics of Emerging Infectious Diseases, Nanchang Center for Disease Control and Prevention, Nanchang, China; ^3^ Department of Microbiology and Immunology, Hamamatsu University School of Medicine, Hamamatsu, Japan; ^4^ The Jiangxi Province Key Laboratory for Diagnosis, Treatment and Rehabilitation of Cancer in Chinese Medicine, Cancer Research Center, Jiangxi University of Chinese Medicine, Nanchang, China

**Keywords:** lung cancer, Merkel cell polyomavirus, Merkel cell carcinoma, cellular tropism, diagnosis, therapeutic strategies

## Abstract

Lung cancer (LC) has been recognized as the leading cause of cancer mortality on a global scale. Although tobacco smoking is predominantly associated with LC (~85%), approximately 15-25% of lung cancers occur in non-smokers. This suggests that other biological cofactors, such as chronic infection and inflammation, are responsible for lung carcinogenesis. Due to the histological similarities between LC and Merkel cell polyomavirus (MCPyV)-associated Merkel cell carcinoma (MCC), studies have investigated their association, particularly small cell lung cancer (SCLC) and non-small cell lung cancer (NSCLC). However, the association between MCPyV infection and LC has been controversial due to inconsistent clinical findings in limited number of cases. To our knowledge, MCPyV is generally ubiquitous and maintains lifelong latent infections in immunocompetent individuals. Thus, its association with high-morbidity cancers raised concerns, and controversy about its cellular tropism as well. Further research is needed to elucidate the pathways by which MCPyV participates the development and progression of LC. Moreover, understanding the role of MCPyV in LC may lead to novel therapeutic strategies. In this review, we critically evaluate the available evidence for and against the aetiological association of MCPyV and LC to help understand its aetiological role, which will provide valuable insights for the diagnosis and therapy of LC.

## Graphical Abstract

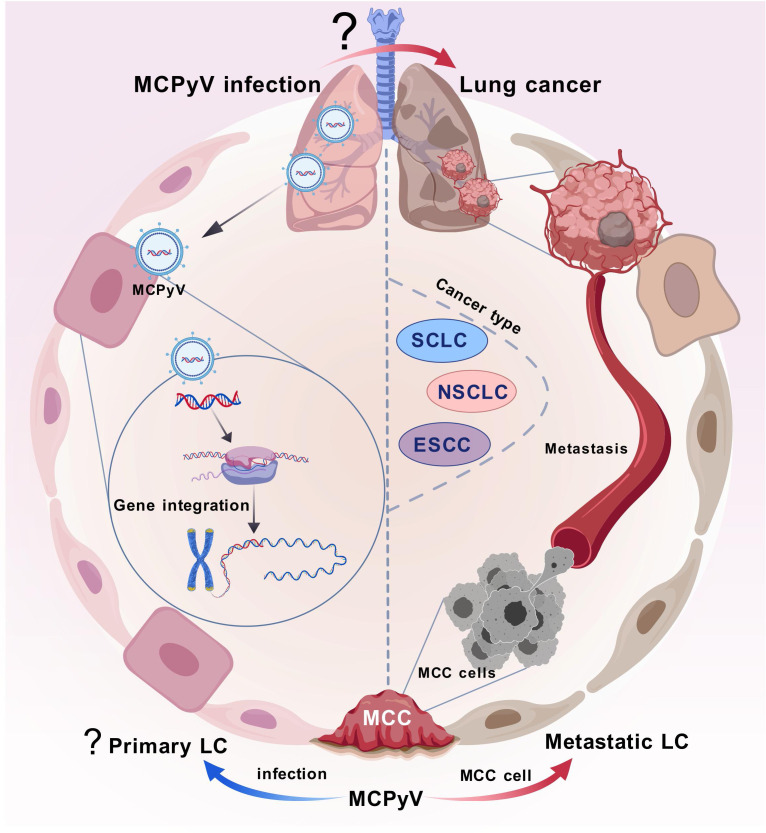

## 1 Introduction

**Figure 1:**
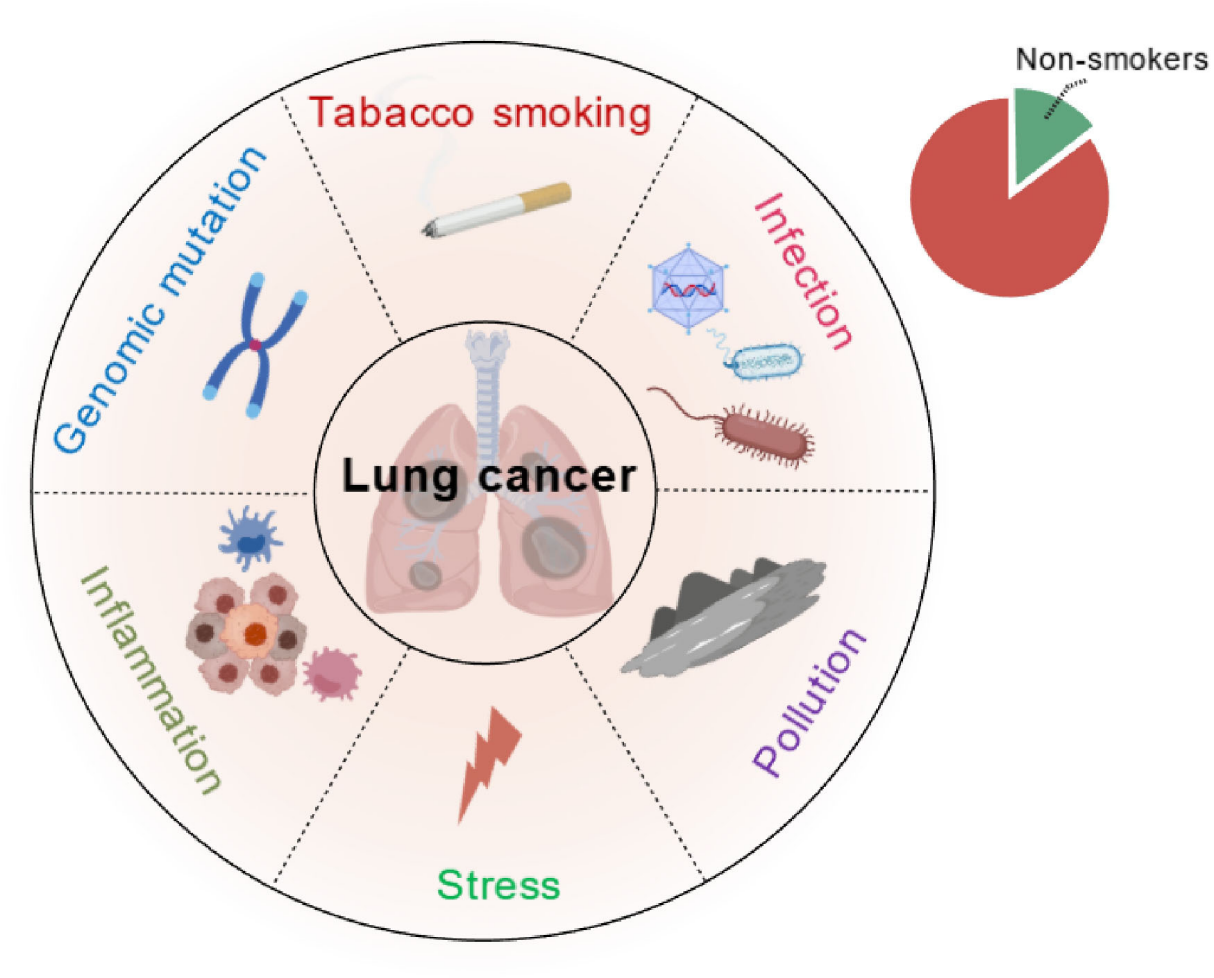
Major potential risk factors of lung carcinogenesis. Smoking includes active smoking and secondhand smoke in non-smokers; Genetic predisposition may play a role, particularly in early-onset cases; Pollution includes hazards exposures such as PM2.5 and arsenic (drinking water); Stress also plays a key role in dysregulating the immune system and promoting systemic inflammation; Viral, bacterial or fungal infections directly or indirectly contribute to lung cancer through chronic inflammation, immune modulation and even direct carcinogenic effects.

Lung cancer (LC) has been recognized as the leading cause of cancer mortality globally. Tobacco smoking is predominantly (~85%) linked to LC, but other risk factors such as air or water pollution, genetic mutation, infections, inflammation and psychological distress are associated with lung carcinogenesis (**Figure 1**) (1–5). To our knowledge, Viruses have played a pivotal role in contemporary cancer research, offering researchers profound insights into the etiology of cancer (6). It has been estimated that approximately 12% of human cancers are associated with viral infection such as Epstein-Barr virus (EBV)-associated nasopharyngeal carcinoma, human papillomavirus (HPV)- associated cervical cancer, hepatitis B virus (HBV)-associated hepatocellular carcinoma, hepatitis C virus (HCV)- associated hepatocellular carcinoma, herpesvirus-associated Kaposi’s sarcoma and Merkel cell polyomavirus (MCPyV)-associated Merkel cell carcinoma (MCC) (7).

Merkel cell polyomavirus (MCPyV) was first identified in 2008 through its detection in tissue samples of Merkel cell carcinoma (MCC), a rare and aggressive neuroendocrine skin cancer (8). It was subsequently classified as the fifth known human polyomavirus and remains the only member of its family established as a primary causative agent of a human cancer (9, 10). Approximately 80% of MCC cases are associated with MCPyV infection, while the remaining cases are largely attributed to UV radiation-induced mutagenesis (11). Multiple studies have reinforced the strong link between MCPyV and MCC. Kassem et al., for instance, detected the virus in a significant proportion of MCC tissues, supporting its potential role in carcinogenesis (12). Duncavage et al. reported MCPyV presence in all 41 examined MCC cases, further underscoring its high prevalence in this malignancy (13). Moreover, Koljonen et al. highlighted an association between chronic lymphocytic leukemia (CLL) and MCPyV-positive MCC, suggesting that immunocompromised individuals such as CLL patients are at increased risk of developing the virus-associated cancer (14). This connection emphasizes the importance of immune status in the progression of MCPyV-related oncogenesis.

Serological studies indicate that MCPyV infection is widespread, with high anti-MCPyV IgG seroprevalence observed across diverse populations (10, 15). The mode of transmission remains under investigation, but evidence from Goh et al. suggests potential respiratory shedding, as MCPyV was detected in respiratory tract secretions (16). This respiratory tropism has prompted research into the possible involvement of MCPyV in lung carcinogenesis, particularly in cancers with neuroendocrine features such as small cell lung cancer (SCLC) and non-small cell lung cancer (NSCLC) (17–21). The histological and behavioral similarities between MCC and SCLC, as noted by Andres et al., further motivate this line of inquiry (22).

SCLC exhibits histological genetic features to MCC, both are of neuroendocrine origin (23). The similarity prompts inquiries into the potential role of MCPyV in the development of SCLC and the mechanisms underlying lung carcinogenesis. Although researcher have identified classifier genes capable of discriminating MCC from SCLC (24), the current state of knowledge on this subject remains inconclusive. In this review, we critically evaluate the evidence for and against the etiological association of MCPyV and LC to help understand its potential etiological role in LC development.

## 2 MPyV genome integration into tumor cells

**Figure 2:**
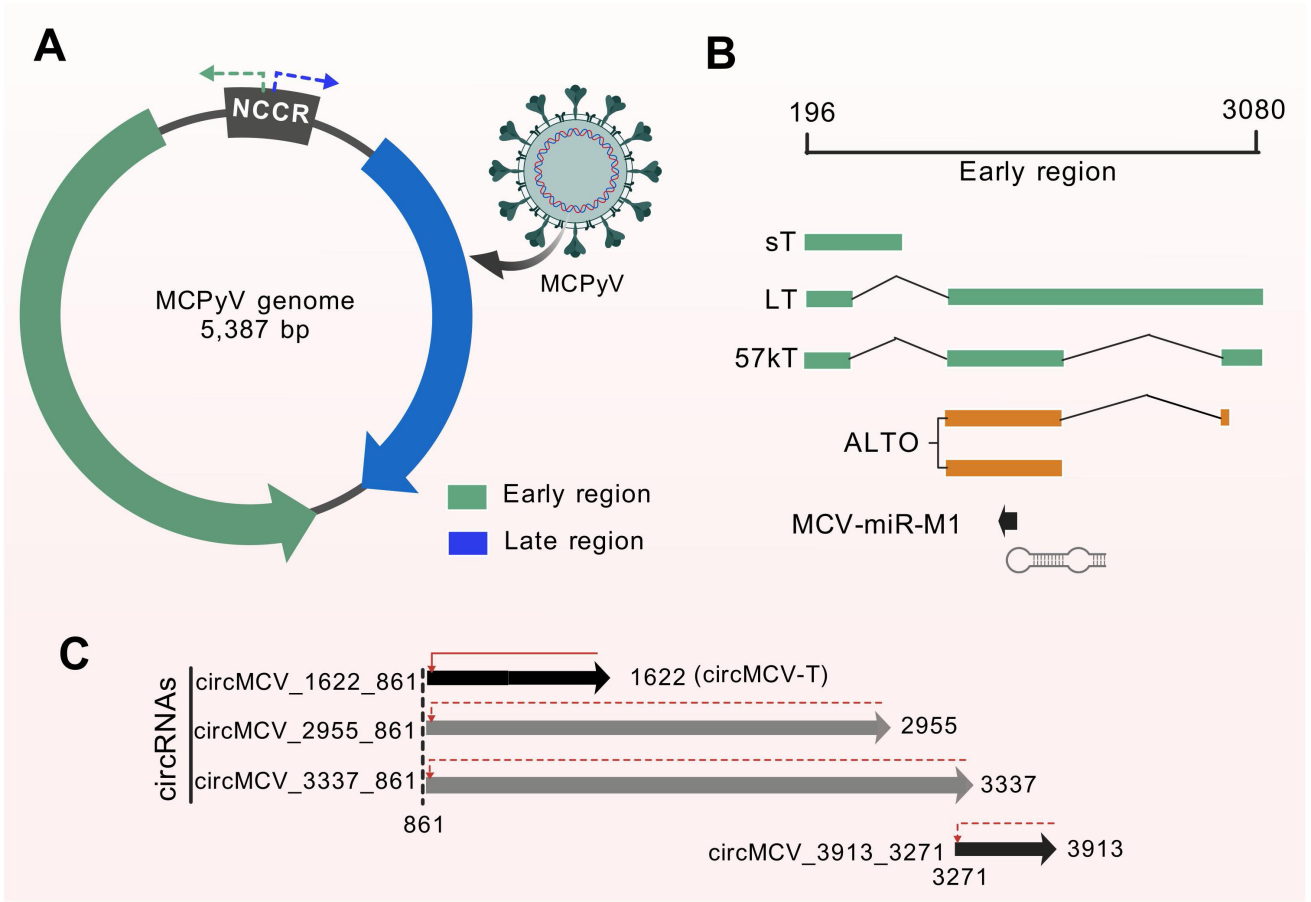
Genome structure of the Merkel cell polyomavirus. **(A)** MCPyV, a double-stranded DNA virus contains a ~5.4 kb circular genome comprising a NCCR, an ER and a LR. **(B)** The ER (196–3080 nt) generates multiple transcripts through a process known as alternative splicing, which involves the use of alternative start sites. These transcripts include LT, ST, 57 kT, the alternative frame of the LT open reading frame (ALTO), and the microRNA MCPyV-miR-M1. **(C)** The figure provides a schematic representation of the four putative MCPyV-encoded circRNA backsplice junctions that have been mapped to the MCPyV-HF genome. The red arrows indicate the direction of the backsplice.

The discovery of MCPyV was made through the digital transcriptome subtraction and sequence analysis of its ~5.4 kb genome (8). MCPyV genome is a double-stranded circular DNA comprising 3 major regions, early region (ER), late region and noncoding control region (NCCR) (**Figure 2A**) (25). The ER codes for two distinct proteins: the early T proteins, also known as the large T antigen (LT), and small T antigen (ST) (26) (**Figure 2B**), and LR codes for viral capsid proteins VP1 and VP2. Recently, Abere et al. identified four putative circRNAs from the ER of MCPyV (**Figure 2C**), underscoring the potential interplay between MCPyV circRNA and miRNA (27). MCV circMCV-T interacts with another MCV noncoding RNA, miR-M1, to functionally modulate early region transcript expression important for viral replication and long-term episomal persistence (27). The presence of MCPyV has been detected in the genomes of up to 90% of human MCC tissues. Research has demonstrated that mutations in the MCPyV T antigens are exclusive to human tumors, exhibiting no impact on the binding of the retinoblastoma tumor suppressor protein. However, these mutations have been observed to result in the elimination of viral DNA replication capacity (28). The expression of MCPyV LT has been detected in the nuclei of tumor cells from MCC, with an average of 5.2 LT DNA copies/cell. Furthermore, it has been demonstrated that the presence of an intact retinoblastoma protein-binding site in MCPyV LT is a prerequisite for the proliferation of MCC cells (29). Improved detection methods have suggested that nearly all MCC tumors harbor MCPyV, with LT expression detected in 97% of unique MCC tumors. Shuda et al. demonstrated that MCPyV-positive MCCs require viral ST for cell proliferation, highlighting the oncogenic potential of viral proteins in driving tumor growth (30). Further research has indicated that MCPyV ST functions as an oncoprotein targeting the 4E-BP1 translation regulator (31). In conclusions, research on the Merkel cell polyomavirus genome structure has provided valuable insights into its integration into human tumors, the role of T antigens in viral replication and tumor growth, as well as potential vaccine development strategies.

## 3 Clinical findings of MCPyV in lung cancer specimens

SCLC has been shown to exhibit similar histological features to MCC (22), The question of whether MCPyV is the causative agent of either SCLC or NSCLC is a matter of some debate within the community. After the identification of MCPyV from MCC tissues, its connection with LC has been explored in many countries using molecular immunology techniques as listed in **Table 1**. Studies from a number of countries have detected viral DNA sequences in LC tissues. Among these studies, researchers observed 4.65%-39 of MCPyV infection positivity in SCLC or NSCLC at the gene or protein levels (17) (**Table 1**). Hashida et al. provided the initial evidence of the detection of MCPyV DNA, as well as the expressions of both LT and its RNA transcripts in NSCLCs (26). The study revealed that nine out of 32 cases of squamous cell carcinoma (SCC), nine out of 45 cases of atypical carcinoid tumor (AC), one out of 32 cases of large-cell carcinoma (LCC), and one out of three cases of pleomorphic carcinomas were positive for MCPyV DNA. the levels of MCPyV LT DNA load among patients with different stages of NSCLC. This finding suggests that as tumors progress, viral load may concomitantly increase (26). A rare case was reported in which a 82-year-old male patient undergoing nivolumab treatment for lung adenocarcinoma subsequently developed Merkel cell carcinoma (MCC). This observation suggests the presence of shared risk factors, including a history of MCPYV infection (17). In a very recent study from South Korea, Jin et al, explored the detection of MCPyV from South Korean patients of NSCLC and found that MCPyV positivity was 22.7% (34 of 150) in NSCLC tissues and 8.0% (12 of 150) in the adjacent tissues (32). Nevertheless, there was a high incidence of HPV coinfection with MCPyV (26.5%, 9 out of 34). Therefore, it is hard to quantify the contribution of MCPyV on NSCLC as HPV is an important causative factor associated with NSCLC.

**Table 1:** Studies on MCPyV detection from LC specimens around the globe (2009-2024).

Year	Country	Case no.	Cancer	Specimen	Target gene	Methods	Positive rate
2009	England (22)	30	SCLC	Tissue	LT, ST	PCR, SB	7.50%
2009	Germany (21)	18	SCLC	Tissue	LT, ST	PCR	39%
2010	USA (33)	30	NSCLC	Tissue	Genome	Sequencing	16.70%
2012	Chile (20)	86	NSCLC	Tissue	LT	PCR	4.65%
2013	Greece (19)	110	NSCLC	Tissue	LT	PCR	9.09%
2013	Japan (34)	112	NSCLC	Tissue	LT	PCR	17.90%
2013	USA (35)	16	ESCC	Tissue	Genome	PCR	18.75%
2014	Iran (36)	78	SCLC, NSCLC	Tissue	VP2	PCR	0%
2014	China (37)	181	NSCLC	Tissue	LT	PCR	17.68%
2016	USA (38)	90	NSCLC	FFPE	LT	IHC	0%
2017	Iran (17)	95	NSCLC	FFPE	LT	PCR	38.95%
2017	Korea (18)	167	NSCLC	FFPE	LT	PCR	17.96%
2017	Germany (39)	MM	Metastasis	Tissue	ST	PCR	NA
2017	Italy (40)	5	Lung tissue	FFPE	LT, VP1	PCR	100%
2017	Bulgaria (41)	88	CLD, LC	Biopsy	VP1	PCR	0%
2018	Italy (42)	1	NSCLC	Biopsy	/	IHC	NA
2024	Korea (32)	150	NSCLC	Tissue	LT, VP1,	PCR	22.70%

MM, mouse model; SCLC, small cell lung carcinoma; NSCLC, non-small cell lung carcinoma; ESCC, extrapulmonary small cell carcinoma; CLD, chronic lung disease; LC, lung cancer; FFPE, Formalin-Fixed Paraffin-Embedded; PCR, polymerase chain reaction; SB, southern blot; IHC, immunohistochemistry; NA, not applicable.

## 4 Metastases of MCC

**Figure 3:**
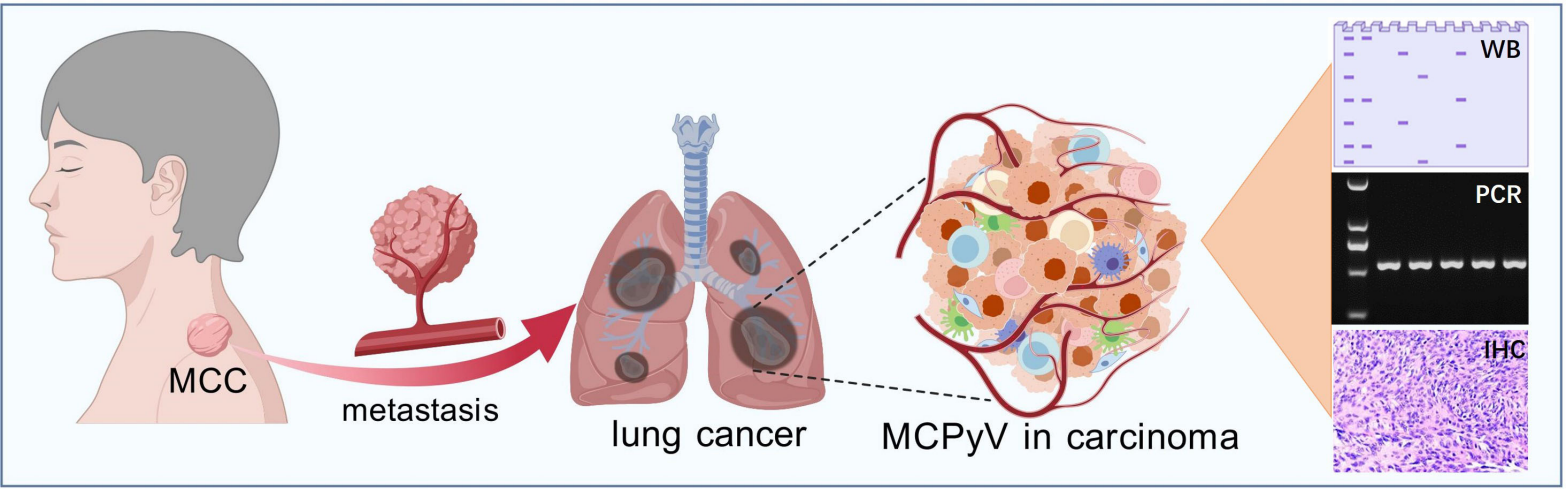
Potential distant metastasis of MCPyV-positive MCC to lung. Integration of multiple detection methods and sequencing technology could efficiently detect MCPyV from carcinoma tissue.

MCC exhibits a notable tendency for regional and distant spread (43). Locally, the tumor often infiltrates surrounding tissues and regional lymph nodes (44). The lymphatic system serves as a primary route for dissemination, with metastases frequently involving regional nodal basins. Clinically palpable lymphadenopathy is a common presentation indicative of regional metastasis (45). Beyond regional nodes, MCC can disseminate hematogenously, leading to distant metastases in organs such as the liver, lungs, bones, and brain. These distant metastases are associated with a markedly poorer prognosis and often present in advanced stages of the disease (43). The aggressive nature of MCC and its capacity for early metastasis are attributed to its neuroendocrine origin and high proliferative index. Tumor cells can invade lymphatic vessels and blood vessels, facilitating dissemination. Molecular factors, including alterations in cell adhesion molecules, matrix metalloproteinases, and neuroendocrine markers, contribute to the invasive potential of MCC (46). Additionally, immune evasion mechanisms—such as the expression of immune checkpoint molecules—may enable tumor cells to survive and establish metastatic sites (47, 48). Nevertheless, study of lung metastases of MCC is limited. Knips et al. found that two animal models with MCPyV-positive MCC cell lines, WaGa and MKL-1, formed lung metastases. These metastases may be driven by ST. Furthermore, the authors observed that, compared to parental cell lines, explanted tumors exhibited an upregulation of MCPyV ST expression (39). Besides, Lewis et al. characterized the metastases in 215 MCC patients and found 7% initial distant metastases occurred in lungs. Among the 215 MCC tissues, 109 had known MCPyV status and 94 (86.2%) are MCPyV-positive (43). Based on this study, we believe that MCPyV can be detected in lungs with MCPyV-positive MCC metastases using integrated molecular diagnostics (**Figure 3**).

Metastasis in Merkel cell carcinoma presents a formidable challenge due to its aggressive behavior and propensity for early dissemination. Understanding the patterns and mechanisms underlying MCC metastases is essential for optimizing diagnostic approaches, refining staging systems, and improving therapeutic strategies. Continued research into the molecular pathways driving metastasis and the development of targeted therapies hold promise for enhancing outcomes in this formidable malignancy.

## 5 Potential pathways of MCPyV involvement of LC carcinogenesis

While genomic integration of MCPyV and subsequent sustained production of LT/ST oncoproteins are well-established drivers of MCC through distinct transformation mechanisms (49), the potential involvement of this polyomavirus in NSCLC pathogenesis remains poorly characterized. Current evidence does not substantiate viral genome integration as a primary oncogenic mechanism in NSCLC development. Emerging research instead points to alternative molecular interactions, particularly involving key regulatory pathways (50). Notably, comparative analyses of MCPyV-positive versus negative NSCLC specimens have revealed a dual pattern of BRAF overexpression and suppressed Bcl-2 expression (19) - a dysregulation profile potentially linked to viral presence. This pathway disruption correlates with observed epigenetic modifications, including altered expression profiles of NSCLC-relevant microRNAs (miR-21, miR-376, miR-145) and their downstream targets, suggesting virus-mediated epigenetic reprogramming (51). Epidermal growth factor receptor (EGFR) mutational patterns further complicate this relationship. Clinical data from Chinese NSCLC cohorts demonstrate significant MCPyV-EGFR mutation co-occurrence, proposing a potential viral role in EGFR mutagenesis (37). This association could mechanistically explain BRAF pathway activation given its position downstream of EGFR signaling. However, genomic mapping studies have identified viral integration sites at 5q23.1 and 11q25 loci24, which lack physical proximity to the EGFR locus at 7p12 (26). This spatial dissociation challenges direct integration-mediated mutagenesis hypotheses, necessitating further investigation into alternative mechanisms of viral-epithelial interaction.

The current NSCLC research paradigm continues to prioritize characterization of canonical driver mutations and immune checkpoint dynamics. Nevertheless, these emerging viral correlations highlight the need for expanded molecular profiling approaches that integrate pathogen-host interaction models with traditional oncogenic pathway analyses.

## 6 Conflicting evidence on the role of MCPyV in LC development

The role of Merkel cell polyomavirus (MCPyV) in lung cancer (LC) development remains controversial, with detection rates ranging from 0% to 39% across different studies (**Table 1**), indicating unclear pathogenicity. However, metastasis of MCPyV-positive Merkel cell carcinoma (MCC) to lung tissue represents a potential pathway, as supported by animal modelsl (39). Several studies have failed to detect MCPyV in lung tumors. Bittar et al. evaluated MCPyV large T antigen (LT) expression via immunohistochemistry (IHC) in 90 lung adenocarcinomas and found no positive cases (38), highlighting the need for molecular studies on viral DNA integration to clarify its role. Similarly, Karimi et al. detected no MCPyV DNA using real-time PCR in 50 SCLC and 28 NSCLC specimens (36). Shikova et al. reported MCPyV DNA in 8.2% of patients with acute respiratory diseases, but found no positivity in nonmalignant chronic lung diseases or lung cancer (41). Furthermore, a study by Stebbing et al. found no association between polyomavirus infections and NSCLC in HIV/AIDS patients, suggesting no clear link between MCPyV and LC development (52).

In contrast, some reports suggest a potential association in specific contexts. Gheit et al. identified MCPyV in NSCLC cases from Chile (20), pointing toward a population-specific risk. Hourdequin et al. also detected MCPyV in extrapulmonary small cell carcinoma, which shares histological features with MCC, implying a broader oncogenic role beyond the skin (35). Nevertheless, seroepidemiological evidence remains inconclusive. Malhotra et al. found no significant difference in polyomavirus seroprevalence between lung cancer cases and controls (53), indicating a complex relationship between infection and cancer risk. Prado et al. provided an overview of human polyomaviruses and their possible roles in oncogenesis, reflecting ongoing interest in the field (54). In conclusion, while some evidence suggests that MCPyV may contribute to lung cancer—either as a primary factor or through metastatic spread—multiple studies have failed to establish a consistent association (43, 55). Further research is essential to elucidate the etiology and underlying mechanisms of MCPyV in lung carcinogenesis.

## 7 Discussion

MCC is a highly lethal cutaneous cancer, and the role of MCPyV in its development has been a subject of investigation (56).The potential involvement of MCPyV in LC pathogenesis remains a subject of intense debate and scientific inquiry. A recent meta-analysis also suggests that MCPyV infection is a potential risk factor for lung cancer (57). While MCPyV is a well-established oncogenic driver in Merkel cell carcinoma (MCC)—a neuroendocrine tumor of the skin—its role in lung carcinogenesis is less clear and fraught with contradictory evidence. Based on the current literature, we posit that MCPyV may contribute to a subset of lung cancers, particularly those with neuroendocrine features such as small cell lung cancer (SCLC), through both direct and indirect mechanisms. However, the available data do not yet support a definitive causative role, and several methodological and biological challenges complicate the interpretation of existing studies.

One major challenge is the considerable variability in MCPyV detection rates across different geographic regions and histological subtypes of LC, as summarized in **Table 1**. Discrepancies may arise from differences in sample processing, sensitivity of detection methods, viral load, and genetic variability of MCPyV strains (32). Furthermore, the ubiquitous nature of MCPyV seroprevalence in the general population complicates the distinction between casual presence and etiological significance. Co-infections with other oncogenic viruses, such HPV, also obscure the individual contribution of MCPyV to tumorigenesis (33). Another layer of complexity arises from the mechanisms through which MCPyV might influence lung carcinogenesis. In MCC, viral integration and continuous expression of LT and ST antigens are critical for oncogenesis (8, 58). In lung cancer, however, such clear mechanistic evidence is lacking. While some studies report MCPyV DNA integration and oncoprotein expression in NSCLC samples, others fail to detect the virus altogether. Alternative pathways—such as viral-induced epigenetic modulation, immune evasion, or interaction with host mutations such as EGFR (37)—may be at play and warrant deeper investigation. The phenomenon of metastatic spread from MCPyV-positive MCC to the lungs further complicates the narrative. Such cases highlight the need to differentiate between primary MCPyV-driven lung tumors and metastatic MCC, which requires meticulous pathological and molecular characterization.

In light of these challenges, we recommend that future studies adopt multi-modal detection strategies—combining DNA, RNA, and protein-based assays—and employ next-generation sequencing to assess viral integration sites and load. Larger, multi-center cohorts stratified by histology, geography, and immune status are essential to clarify the virus’s role. Moreover, functional studies in models of lung epithelium are needed to elucidate whether MCPyV can directly transform bronchial or alveolar cells.

In conclusion, while there is intriguing evidence suggesting that MCPyV may contribute to certain types of lung cancer, particularly in the context of specific genetic or immune backgrounds, current data are insufficient to establish a firm causal link. Overcoming the existing research hurdles will not only clarify the oncogenic potential of MCPyV in the lung but may also reveal novel therapeutic targets for a subset of lung cancer patients.
